# Feature-aware unsupervised lesion segmentation for brain tumor images using fast data density functional transform

**DOI:** 10.1038/s41598-023-40848-5

**Published:** 2023-08-21

**Authors:** Shin-Jhe Huang, Chien-Chang Chen, Yamin Kao, Henry Horng-Shing Lu

**Affiliations:** 1 Geometric Data Vision Laboratory, Department of Biomedical Sciences and Engineering, National Central University, Taoyuan City, 32001 Taiwan; 2 Institute of Statistics, National Yang Ming Chiao Tung University, Hsinchu, 30010 Taiwan

**Keywords:** Biophysics, Medical research

## Abstract

We demonstrate that isomorphically mapping gray-level medical image matrices onto energy spaces underlying the framework of fast data density functional transform (fDDFT) can achieve the unsupervised recognition of lesion morphology. By introducing the architecture of geometric deep learning and metrics of graph neural networks, gridized density functionals of the fDDFT establish an unsupervised feature-aware mechanism with global convolutional kernels to extract the most likely lesion boundaries and produce lesion segmentation. An AutoEncoder-assisted module reduces the computational complexity from $\mathcal{O}\left({N}^{3}\right)$ to $\mathcal{O}\left(N\mathrm{log}N\right)$, thus efficiently speeding up global convolutional operations. We validate their performance utilizing various open-access datasets and discuss limitations. The inference time of each object in large three-dimensional datasets is 1.76 s on average. The proposed gridized density functionals have activation capability synergized with gradient ascent operations, hence can be modularized and embedded in pipelines of modern deep neural networks. Algorithms of geometric stability and similarity convergence also raise the accuracy of unsupervised recognition and segmentation of lesion images. Their performance achieves the standard requirement for conventional deep neural networks; the median dice score is higher than 0.75. The experiment shows that the synergy of fDDFT and a naïve neural network improves the training and inference time by 58% and 51%, respectively, and the dice score raises to 0.9415. This advantage facilitates fast computational modeling in interdisciplinary applications and clinical investigation.

## Introduction

The rise of computer vision technology has delineated a macroscope technical blueprint for biometric identification nowadays. Sophisticated deep neural networks on lesion recognition, tracking, and segmentation of various medical images have also further promoted the evolution of clinical investigations. Glioma modality identification has attracted significant attention from scientists and engineers and orientated the mainstream development of tumor image segmentation due to its high aggressiveness^1,2^ and infiltrative^3^ properties. Thus, image-vision-based neural networks have reached fruitful achievements on high-dimensional and multi-channel glioma image recognition and segmentation tasks^3,4^. However, neural networks are pushed to incorporate more complex structures and require large datasets and time costs to train the model effectively. Complicated and non-unified deep network networks also make optimizing the model structure difficult, increase hardware costs, and reduce the execution efficiency of embedded computing units. Fortunately, the emergence of geometric deep learning (GDL) technology is opening up an avenue for these problems.

By considering the intrinsic geometric configuration of image textures, the GDL methods map them into a non-Euclidean space for feature searching. Supported by its theoretical framework, which is based on physics, both the structure and the operation of neural networks become explainable^5^. Adopting transformation symmetry and invariance increases the identification capability of neural network models for graphic representation, thereby significantly reducing the dependency of the deep learning model on large training sets^6^. But it turns the problem to analyzing complex differential geometry in non-Euclidean spaces^7^. The solution to this difficulty is to use the geometric properties of the image textures in terms of their physical behavior in Euclidean space. To simultaneously consider the medical image textures, pixel intensity distributions within medical images, and the interactions between pixel pairs, introducing the energy-mapping method based on the density functional theory (DFT) may be a good starting point^8^.

The modern DFT and contemporary deep neural networks share the demand for an initial guess and iteration, which speeds up their fusion and synergic effect. The initial guess of electron density functions is essential in iterations of DFT frameworks. It also benefits the procedure of supervised backpropagation for training physical parameters in deep learning approaches^9,10^. From DFT’s perspective, the random initial parameter would have definite meanings bestowed by the intrinsic essence of many-particle systems. Relevant studies of pattern recognition across scientific applications have exhibited successful combinations of DFT and deep neural networks, including density functional training models^11,12^, the composition and morphology of nanoparticle surface textures^13^, configurations of high-dimensional electron density and energy functionals^9,14,15^, the unsupervised clustering of compounds^16^, and simulations and modelings of complex systems^17–22^. Although incorporating DFT with these data learning methods has led to a research trend in materials science, the heterogeneity in their data structures still impedes configurational integration.

Machine learning methods like multilayer perceptrons^12,14,15,17^ and unsupervised clustering^16–21^ are still mainstream compared to image-vision-based neural networks, such as convolutional neural networks (CNNs)^9,11,14,15^, mainly attributed to the low-dimensional data structures of DFT framework. However, valuable information from high-dimensional data structures like the energy landscapes^20,21^ or atomic configurations^21,22^ may be missed. Introducing the GDL’s concept is also obstructed^5^. On the other hand, most neural network layers and activation functions have to be manually modulated. Despite initial guess training possessing representative physical meanings, it isn’t easy to reflect the configuration of a many-particle system with complex textures when incorporated with neural networks. For instance, node numbers in multilayer perceptrons and sizes of convolutional kernels in CNNs directly affect the construction of the receptive field on data morphology. Neural networks combine or transform the trained parameters extracted from those receptive fields to establish corresponding feature maps. Since these operations do not consider the long-range dependency between data points and scale the dimensions of the data morphology, the feature maps generated could not reveal detailed textures of an actual physical system. Hence, energy landscapes or high-dimensional atomic structures reconstructed by these feature maps might lose the original physical essence. In recent investigations, energy-based neural networks in pattern recognition tasks are pursuing better frameworks to solve this predicament by estimating the pair-wise dependency of image pixels with a Coulombic-like form^23–25^, optimizing neural network structures^26^, constructing energy landscape of medical images^8^, or connecting data points using density functionals^27^. However, these network frameworks still have to burden the high computational complexity^27^, large memory storage^24^, and heavy data transmission loads^26^.

**Figure 1:**
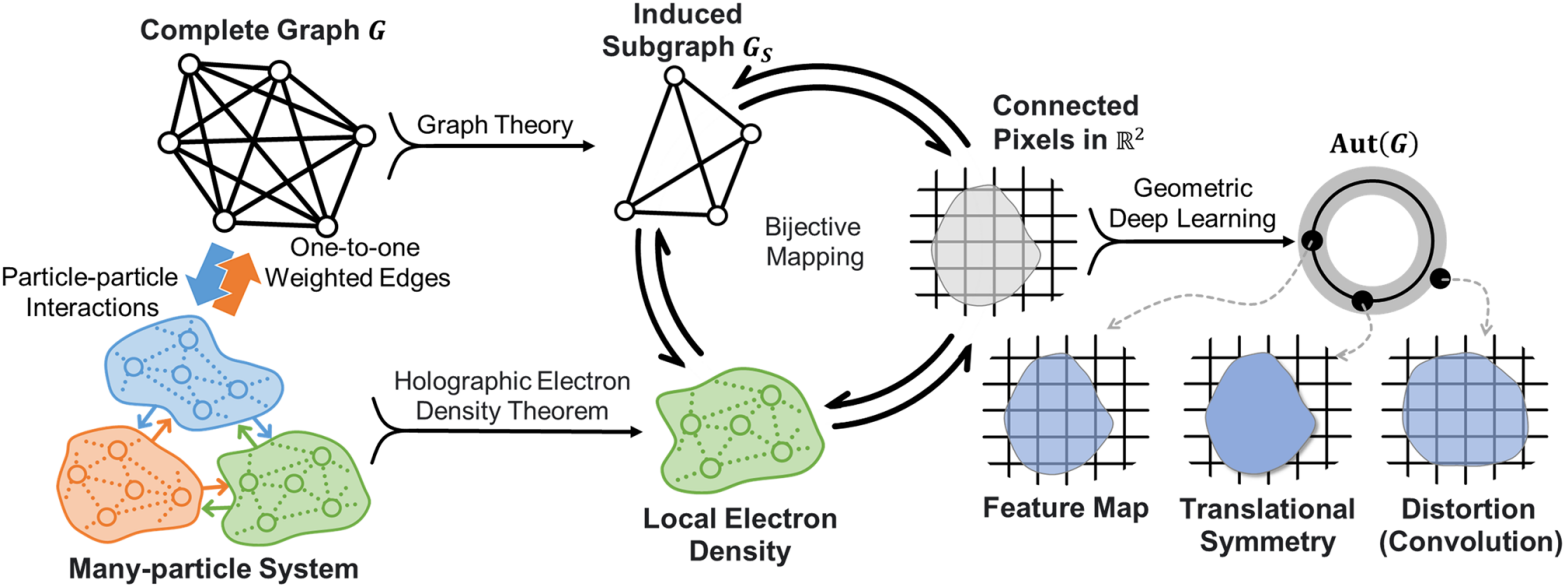
The corresponding relationship between a many-particle system, a complete graph, and an image grid space ${\mathbb{R}}^{2}$. Geometric deep learning establishes the formation of a set of connected pixels to a feature map in an automorphic group. We only delineated the interactions (the dashed lines) within pair nodes in the many-particle system to avoid complicated structures occurring between them.

To integrate the data structures from DFT and image-vision-based neural networks without bearing these engineering problems, we propose fusing the holographic electron density theorem and the GDL architectures. The holographic electron density theorem^28^ describes that once we obtain an electron density function within any finite volume of a many-particle system, we know the whole configuration of density functions inside this system in principle. By introducing this concept into the framework of graph neural networks (GNNs), we first assume that the configuration of a local electron density has bijective mappings (isomorphism) in a graph structure $G=\left({\mathcal{V}}_{G},{\mathcal{E}}_{G}\right)$, with a vertex set ${\mathcal{V}}_{G}$ and an edge set ${\mathcal{E}}_{G}$, and can construct a topological subgraph ${G}_{S}=\left({\mathcal{V}}_{{G}_{S}},{\mathcal{E}}_{{G}_{S}}\right)$ of $G$, where ${\mathcal{V}}_{{G}_{S}}\subseteq {\mathcal{V}}_{G}$ and ${\mathcal{E}}_{{G}_{S}}\subseteq {\mathcal{E}}_{G}$. Thus we infer that the given topological subgraph ${G}_{S}$ can determine all attributes of the topological graph $G$ when each vertex in $G$ has one-to-one weighted edges with all other ones, similar to the holographic electron density theorem. In other words, $G$ is a complete graph, and ${G}_{S}$ is an induced subgraph of $G$. The one-to-one weighted edges represent the particle-pair interactions in a physical system, while a topological subgraph indicates a local electron density. Embedding the topological graph $G$ into two-dimensional Euclidean space ${\mathbb{R}}^{2}$, the graphic structure reduces to a specific matrix, for instance, a medical image in grid space. Thus we can treat connected pixels of medical images as physical particle clusters and topological subgraphs simultaneously under the GNN metrics and use them to estimate corresponding density functionals through the DFT or extract feature maps from CNNs. Figure 1 illustrates their complementary relationship.

On the other hand, the GDL treats these feature maps as geometric priors, and the operations of convolutional kernels and image pooling correspond to translational symmetry and scale separation^5^, respectively. Since sets of bijective mappings from the feature maps in Euclidean space onto itself form an automorphic group $\mathrm{Aut}\left(G\right)$, we can always define a symmetry group from it to inspect the properties of transformation invariance or equivariance^5,29^ of the feature maps from CNNs. It means the feature maps and the results obtained from convolutional and pooling operations belong to or are around the same symmetry group. These properties then benefit the theoretical combination of the DFT and CNNs under the GDL architecture and GNN metrics.

In the present work, we rebuilt the frameworks of the DFT and CNNs in the manner mentioned above for unsupervised lesion recognition and segmentation of brain tumor images. We showed that the fast data density functional transform (fDDFT)^4^ achieves the desired theoretical combination and structural integration between the DFT and convolutional operations. Kinetic and potential energy density functionals (KEDF and PEDF) under the fDDFT framework provide the procedures for intensity enhancement and global convolutions of input medical images, respectively. According to the definition of the automorphic group under the GDL architecture, the input image and the KEDF and PEDF landscapes belong to the same symmetry group since these energy landscapes are equivariant to the transformation of the input image^5^. Disjoint subsets of the pixels, namely orbits, allow us to group them based on specific structural rules intuitively^29^. The group of these subsets forms the feature maps from CNN’s perspective. The linear combination of the KEDF and PEDF determines the Lagrangian density functional (LDF) in the grid space, and rules of structural recognition and segmentation directly rely on the geometric stability of the LDF. Thus these gridized density functionals all have characteristic operations on input medical images in the grid space.

Moreover, we introduced (1) a speed-up scheme by replacing the integral of PEDF of DDFT^8^, a previous version of the fDDFT, with global convolutional operations, (2) an energy-based loss function based on the gradient ascent of LDF to resolve geometric instability occurs at grid boundaries, (3) an AutoEncoder-assisted module, an unsupervised neural network, built by the smoothness of the PEDF landscape, (4) a framework of feature-aware unsupervised pattern recognition and segmentation on three-dimensional brain tumor image datasets, and (5) the limitation of the method when suffering the situation of low-featured subgraph representations. To reveal the performance of the unsupervised pattern recognition and segmentation of the proposed framework in the grid system with high-level heterogeneity, we employed gray-level medical image datasets to mimic chaotic physical environments in the study. To verify our method, we estimated the three-dimensional soft dice score for each segmented brain tumor image^30^. In all study cases, we find that the geometric stability of the LDF landscape enhances the effect of brain tumor image recognition, the similarity convergence assists the feature selection on high-dimensional image structures, and the AutoEncoder-assisted module significantly reduces the computational complexity. These results support the success of theoretical combination and structural integration between the DFT and neural networks in this study. However, DFT-based methods have limitations in segmenting low-featured subgraphs embedded in grid space with unstable energy ranges, like those medical image subgraphs with low energy, weak connectivity, and low heterogeneity.

## Methods

Isomorphically mapping the data intensity matrices into energy spaces, the DDFT simultaneously reveals data significance and similarity by computing their KEDF and PEDF, respectively^8,31^. For a $D$-dimensional transformation, the relationship between the intensity matrix $\rho$ and the pseudo-Fermi level $k_{F}$ has the compact form of $\rho \left[ {k_{F} } \right] = k_{F}^{D} /\left[ {D\left( {2\pi } \right)^{D} } \right] \in {\mathbb{R}}^{D}$^8^. Under this relation, the KEDF landscape $t\left[ \rho \right]\left( {\varvec{r}} \right)$ and PEDF landscape $u\left[ \rho \right]\left( {\varvec{r}} \right)$ have the following expressions in a two-dimensional grid space ${\mathbb{R}}^{m \times n}$:

1 $$t\left[\rho \right]\left({\varvec{r}}\right)=2{\pi }^{2}\rho \left({\varvec{r}}\right)\in {\mathbb{R}}^{m\times n}$$

and

2 $$u\left[\rho \right]\left({\varvec{r}}\right)=\frac{1}{2}\int \frac{\rho \left({{\varvec{r}}}^{\boldsymbol{^{\prime}}}\right)}{\left|{\varvec{r}}-{{\varvec{r}}}^{\boldsymbol{^{\prime}}}\right|}{d}^{2}{{\varvec{r}}}^{\boldsymbol{^{\prime}}}\in {\mathbb{R}}^{m\times n}$$

Arguments ${\varvec{r}}\in {\mathbb{N}}^{2}$ and ${{\varvec{r}}}^{\boldsymbol{^{\prime}}}\in {\mathbb{N}}^{2}$ represent position coordinates of an observed point and a source point in the $m\times n$ grid space, respectively. The KEDF landscape represents an enhanced pixel intensity distribution, whereas the PEDF landscape uses a Coulombic potential to estimate long-distance interaction and measure data similarity between pair pixel points.

To compute the LDF and solve the unit mismatch between KEDF and PEDF, the DDFT constructs the LDF landscape $\mathcal{L}\left[\rho \right]\left({\varvec{r}}\right)$ in a scale-free manner:

3 $$\mathcal{L}\left[\rho \right]\left({\varvec{r}}\right)={\gamma }^{2}t\left[\rho \right]-\gamma u\left[\rho \right]\in {\mathbb{R}}^{m\times n}.$$

Similarly, the Hamiltonian density functional (HDF) landscape has the following expression:

4 $$\mathcal{H}\left[\rho \right]\left({\varvec{r}}\right)={\gamma }^{2}t\left[\rho \right]+\gamma u\left[\rho \right]\in {\mathbb{R}}^{m\times n}.$$

The adaptive scaling factor $\gamma$ in the above equations is the core of DDFT. It prevents the imbalance between KEDF and PEDF landscapes when being scaled or normalized:

5 $$\gamma =\frac{1}{2}\frac{\langle u\left[\rho \right]\rangle }{\langle t\left[\rho \right]\rangle }\in {\mathbb{R}}.$$

The measures $\langle u\left[\rho \right]\rangle \in {\mathbb{R}}$ and $\langle t\left[\rho \right]\rangle \in {\mathbb{R}}$ are global means of PEDF and KEDF landscapes, respectively. The LDF landscape is the difference between the regularized KEDF and PEDF landscapes. Hence, points with zero values (i.e., stable locations geometrically) on the LDF landscape reveal the boundaries where the interactions between inside and outside terminate. Disjoint subgraphs enclosed by the boundaries on the LDF landscape have high data significance and similarity and form an aware feature map. Similar situations occur in the medical image matrices. The LDF would label heterogeneous components within the images after a global search of their corresponding boundaries. These components with intensities over the mean level of LDF would become a partition of the aware feature map.

### The framework of the fast data density functional transform (fDDFT)

The typical procedure to estimate the PEDF of image matrices in Eq. (2) is to numerically transfer the integral to a discrete sum, i.e., $\frac{1}{2}{\sum }_{i=1}^{m\times n}\rho \left({{\varvec{r}}}_{i}^{\boldsymbol{^{\prime}}}\right)\Delta {{\varvec{r}}}_{i}^{\boldsymbol{^{\prime}}}/{\Vert {\varvec{r}}-{{\varvec{r}}}_{i}{\prime}\Vert }_{{\varvec{r}}\ne {{\varvec{r}}}_{i}{\prime}}$, and calculate the corresponding values pixel by pixel in order. The computational complexity of $\mathcal{O}\left({N}^{3}\right)$^27^ for consecutively searching image pixels is so high that parallel computations become inevitable^8^. To expedite the PEDF estimation without additional hardware costs, the relationship between the pixel intensity matrix $\rho \left({{\varvec{r}}}^{\boldsymbol{^{\prime}}}\right)$ and the kernel $k\left({{\varvec{r}}}^{\boldsymbol{^{\prime}}};{\varvec{r}}\right)=1/\left(2\left|{\varvec{r}}-{{\varvec{r}}}^{\boldsymbol{^{\prime}}}\right|\right)$ in Eq. (2) was redefined and treated as a convolution in this work:

6 $$u\left[ \rho \right]\left( \varvec{r} \right): = \rho \left( {\varvec{r^{\prime}}} \right) { \circledast } k\left( {\varvec{r^{\prime}};\varvec{r}} \right).$$

The convolution between $\rho \left({{\varvec{r}}}^{\boldsymbol{^{\prime}}}\right)$ and $k\left({{\varvec{r}}}^{\boldsymbol{^{\prime}}};{\varvec{r}}\right)$ in the ${{\varvec{r}}}^{\boldsymbol{^{\prime}}}$ domain equals their product of Fourier transform $P\left( {j\varvec{k^{\prime}}} \right) \cdot K\left( {j\varvec{k^{\prime}}} \right)$ in a $\varvec{k^{\prime}}$ domain, i.e.,$\rho \left( {\varvec{r^{\prime}}} \right) { \circledast } k\left( {\varvec{r^{\prime}};\varvec{r}} \right) \Leftrightarrow P\left( {j\varvec{k^{\prime}}} \right) \cdot K\left( {j\varvec{k^{\prime}}} \right)$. Hence, we established a new approach for the PEDF estimation to replace the complicated integrals by calculating their product in the ${{\varvec{k}}}^{\boldsymbol{^{\prime}}}$ domain, employing zero paddings^32^ for the product, and then inverting back to the ${{\varvec{r}}}^{\boldsymbol{^{\prime}}}$ domain. Under this framework, the computational complexity reduces to $\mathcal{O}\left(N\mathrm{log}N\right)$.

**Figure 2:**
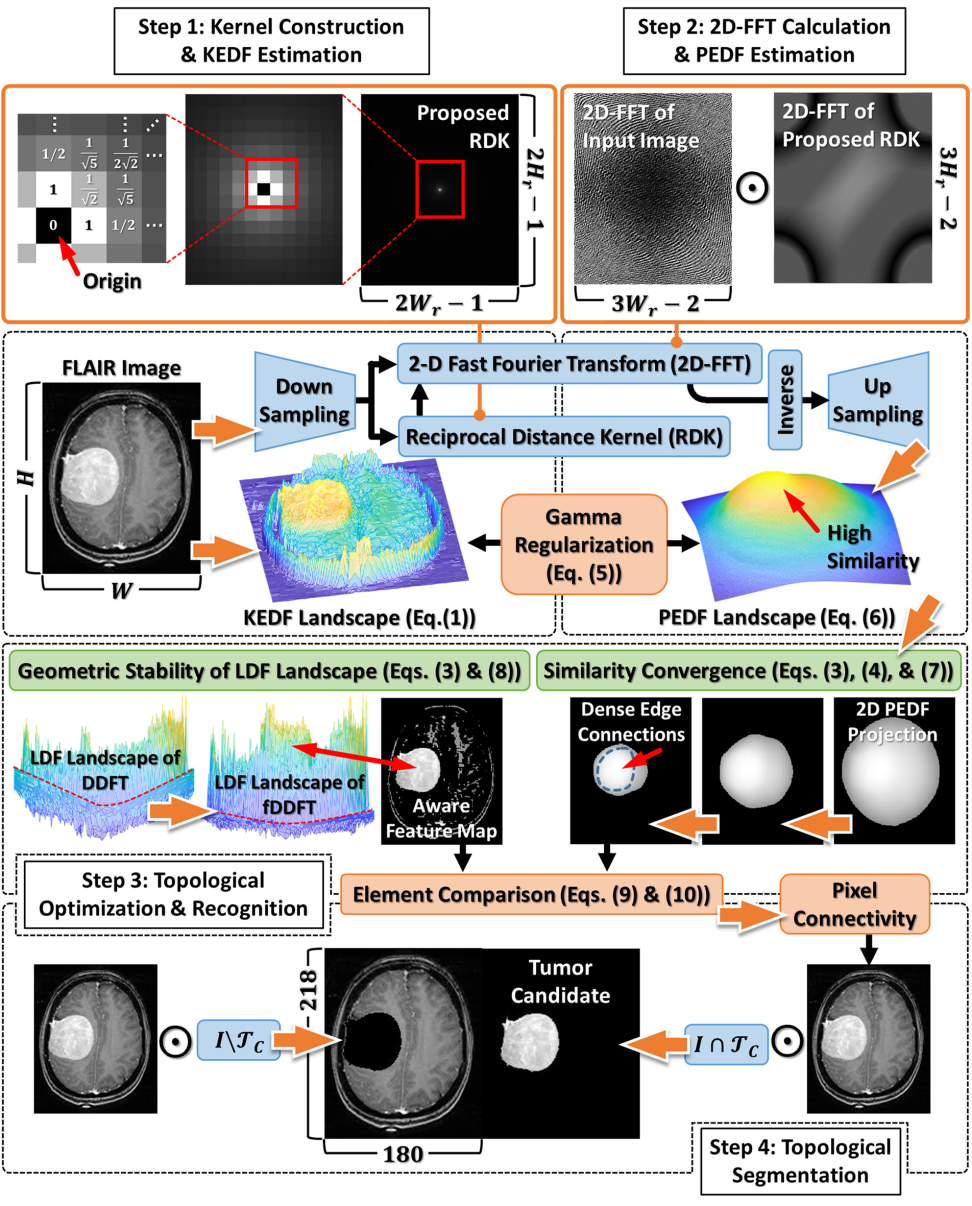
The flowchart of the proposed fDDFT. In Step 1, the mapping of the FLAIR image^33^ constructs the KEDF landscape, followed by forming the reciprocal distance kernel (RDK). In Step 2, element-wise products of 2D-FFTs of the image and kernel are calculated and then inversed for PEDF estimation. In Step 3, two algorithms are introduced to deal with problems of similarity convergence and geometric instability in the PEDF and LDF landscapes, respectively. Comparing elements of the enhanced PEDF landscape and the aware feature map, indicating a specific subset group of the tumor. In the last step (Step 4), segmentation of the candidate tumor image is achieved automatically using the characteristics of pixel connectivity. We provided the relevant algorithms in Supplementary Code.

Figure 2 displays the flowchart of the fDDFT. In Step 1, we first used Eq. (1) to delineate the KEDF landscape of an input brain tumor image $I\in {\mathbb{R}}^{H\times W}$ acquired from an open-source dataset^33^. Then we constructed the kernel $k\left({{\varvec{r}}}^{\boldsymbol{^{\prime}}};{\varvec{r}}\right)$ based on its spatial characteristics in 2-dimensional Euclidean. The values around the kernel origin, indicated by the red arrow in the inset, are the reciprocal offsets of Euclidean distances compared to the origin point. According to this mathematical property, we defined the structure $k\left({{\varvec{r}}}^{\boldsymbol{^{\prime}}};{\varvec{r}}\right)$ as the reciprocal distance kernel (RDK). The dimensional factors of RDK, ${H}_{r}$ and ${W}_{r}$, are products of image dimensions and a dimension-reduced factor, respectively. Due to the smoothness of the PEDF landscape (see Step 2), the dimensions of the input image and its corresponding RDK can be compressed for the following operations and the PEDF estimation. The PEDF landscape can be reconstructed back to its original dimensions without losing information. To apply this advantage to the fDDFT framework, we introduced an AutoEncoder-assisted module^20^ (i.e., the blue blocks) to manage this operation. We found that setting the dimension-reduced factor to 12.5%, i.e., $\left({H}_{r}, {W}_{r}\right)=0.125\times \left(H, W\right)$, did not affect experimental results.

In Step 2, the 2-dimensional fast Fourier transforms (2D-FFTs) of the input image and RDK establish the PEDF landscape by inversing and mapping their element-wise product back onto the gridized energy space. The symbol $\odot$ is the Hadamard product. It should emphasize that the 2D-FFT embedded in the AutoEncoder-assisted module is equivalent to a global convolutional operation. The module with a given dimension-reduced factor could effectively reduce the computational complexity caused by convolutional operations of conventional CNNs. The gamma regularization of Eq. (5) balances the unit mismatch between KEDF and PEDF. On the other hand, since the PEDF landscape represents the similarity between pixel points, it accentuates the subgraphs in grid space that have attributes of high-intensity or dense edge connections, as indicated by the red arrow in the inset. Recent research also validates that multi-view learning could reinforce the complementary information of different views, and the feature-searching in a specific latent space would raise the accuracy of object recognition^3,20^. Thus, this work supports the mechanism of our proposed AutoEncoder-assisted module and the 2D-FFT operations in the latent space.

### The similarity convergence and the geometric stability

Skulls and normal brain tissues with high intensity in the PEDF landscape often affect the capability of tumor recognition and segmentation. To prevent this, in Step 3, we utilized the Fermi normalization to only extract the subgraph ${\mathcal{U}}_{E}$, which has dense edge connections. The Fermi normalization is^30^

7 $$\mathcal{F}\mathcal{N}\left(\rho ;{\rho }_{F},{\rho }_{S}\right)=\frac{1}{{e}^{-\left(\rho -{\rho }_{F}\right)/{\rho }_{S}}+1}\in {\mathbb{R}}^{H\times W}.$$

Parameters ${\rho }_{F}\in {\mathbb{R}}$ and ${\rho }_{S}\in {\mathbb{R}}$ are the global mean and standard deviation of the input matrix $\rho$, respectively. Here the input matrix, in this case, was the PEDF Landscape. Established from the foundation of Fermi–Dirac distribution and the concept of unsupervised learning^30^, the Fermi normalization combines the advantages of z-score normalization and sigmoid activation function. The former $x=\left(\rho -{\rho }_{F}\right)/{\rho }_{S}$ uses ${\rho }_{F}$ as an energy level, i.e., a Fermi-like level of the PEDF landscape, and then ${\rho }_{S}$ to modulate the energy pattern of the PEDF landscape. The latter $1/\left({e}^{-x}+1\right)$, a frequently used activation function in CNNs, squeezes the range into $\mathcal{F}\mathcal{N}\in \left[0, 1\right]$. Thus, the Fermi normalization can search out the data points having intrinsic energy exceeding ${\rho }_{F}$. Moreover, magnet resonance (MR) images generated from different imaging protocols often have different intensity attributes; Fermi normalization can also offer them the same ground state. It makes those pixel elements that should be in the same subgraphs to have similar intensity attributes. To avoid influences from high-intensity pixel elements, we used the Fermi normalization to preserve the elements having the condition of $\mathcal{F}\mathcal{N}>0.5$. Similar to the concept of level set, we continued the procedure until the rest pixel number was less than half of the total data length. We styled this design as the similarity convergence, as illustrated in the sequential insets in Step 3 of Fig. 2.

On the other hand, the border discontinuity of the PEDF landscape results in the geometric deformation of the LDF landscape. As shown in the LDF landscape of the DDFT version in Step 3 of Fig. 2, the problem of this geometric instability, as delineated by the dashed red line with high curvature, would hide elements having high similarity. A suitable adaptive scaling factor is required to alleviate the geometric deformation that occurred in the DDFT version. Inspired by the backpropagation mechanism in neural networks, we introduced a gradient ascent associated with the statistical property of the LDF in the fDDFT framework to update the adaptive scaling factor. The adaptive scaling factor expressed in Eq. (5) becomes:

8 $${\gamma }_{new}\leftarrow {\gamma }_{previous}+\eta \frac{{\langle \mathcal{L}\left[\rho \right]\rangle }_{new}}{{\langle \mathcal{L}\left[\rho \right]\rangle }_{previous}}\in {\mathbb{R}}.$$

Symbols ${\langle \mathcal{L}\left[\rho \right]\rangle }_{new}\in {\mathbb{R}}$ and ${\langle \mathcal{L}\left[\rho \right]\rangle }_{previous}\in {\mathbb{R}}$ represent the global LDF means in new and previous states, respectively. Parameter $\eta$ is the learning rate and is set to 0.5 for convenience. The stopping criterion for updating Eq. (8) is ${\langle \mathcal{L}\left[\rho \right]\rangle }_{new}\ge 0$ to embody the geometric stability in physical structure. The base curvature of the LDF landscape in the fDDFT version of Step 3 in Fig. 2, as delineated by the dashed red line, became more flattened. The elements having high similarity also became significant, as indicated by the red arrow.

The two-dimensional projection of the LDF landscape, updated by the geometric stability algorithm, forms an aware feature map in the grid space, which includes the subgraphs composed of the tumor, the skull, and some brain tissues. Please note that the criterion of ${\langle \mathcal{L}\left[\rho \right]\rangle }_{new}\ge 0$ inhibits the elements in the aware feature map with intensity levels lower than the value of ${\langle \mathcal{L}\left[\rho \right]\rangle }_{new}$. Thus the aware feature map would provide information on the most likely locations and the corresponding boundaries of the brain tumor image. To recognize and segment the subgraphs composed of the tumor elements from the aware feature map, its $N$ observations ${\sum }_{i=1}^{N}{\mathcal{T}}_{{S}_{i}}$ to the subgraph ${\mathcal{U}}_{E}$ of the PEDF landscape must be compared. Once elements in ${\mathcal{T}}_{{S}_{i}}$ and ${\mathcal{U}}_{E}$ have the same spatial coordinates, their belonging subgraphs would become the tumor candidates:

9 $${\mathcal{T}}_{C}=\left\{{\mathcal{T}}_{{S}_{i}}|{\mathcal{U}}_{E}\cap {\mathcal{T}}_{{S}_{i}}\notin \varnothing \right\}, i=\mathrm{1,2},\dots ,N.$$

Through the introduction of pixel connectivity, the candidates of segmented tumor elements and the rest brain tissues belong to the subgraphs of $\left\{I\cap {\mathcal{T}}_{C}\right\}$ and of $\left\{I/{\mathcal{T}}_{C}\right\}$, respectively, as illustrated in Step 4 of Fig. 2. Therefore, brain tumor image elements are automatically recognized and eventually segmented under the proposed fDDFT framework. We provided the relevant algorithms in Supplementary Code.

### Programming implement environment

In the data preprocessing procedure, we normalized the image intensity into the range of [0, 1] to save memory sizes before removing the noise from each original image. Then we recorded the rectangular dimensions of their sub-components using bounding boxes. In the procedure of fDDFT estimation, the dimension-reduced input image and the RDK had a global convolutional operation, and the result was scaled back to its original dimensions to construct the PEDF and KEDF landscapes. The adaptive scaling factor was used to balance the unit mismatch between the PEDF and KEDF, and these regularized functionals composed the corresponding LDF and HDF landscapes. In establishing the geometric stability of the LDF landscape, the procedure continuously updated the adaptive scaling factor and LDF until the global mean of LDF was higher than zero. In the step of similarity convergence, we defined ranges having high similarity by utilizing Fermi normalizations to define two-dimensional projection areas of HDF and LDF landscapes. Then these high-similarity ranges proposed candidates for the subgraphs of aware feature maps in the step of pixel connectivity. Only subgraphs with edges larger than the permissible smallest edge number and with dimensions 75% smaller than that of the bounding boxes could join into the aware feature map. We then sequentially arranged these aware feature maps to establish its three-dimensional brain tumor structure by choosing components with the highest energy level and the largest voxel. Eventually, we employed the three-dimensional soft dice score metric to calculate the accuracy of segmented brain tumor images extracted from the aware feature map^30^:

10 $$DS=\frac{2\sum_{i}^{H\times W\times D}{t}_{i}{g}_{i}}{\sum_{i}^{H\times W\times D}{t}_{i}^{2}+\sum_{i}^{H\times W\times D}{g}_{i}^{2}+\epsilon }\in {\mathbb{R}}.$$

The symbols $H\times W\times D$ and $\epsilon ={10}^{-5}$ are the total grid number in a three-dimensional grid space and a factor to avoid $DS$ diverging, respectively. The parameters ${t}_{i}\in \left\{\mathrm{0,1}\right\}$ and ${g}_{i}\in \left\{\mathrm{0,1}\right\}$ are the binary tumor image segmentation prediction and ground truth labeling, respectively.

## Results

**Figure 3:**
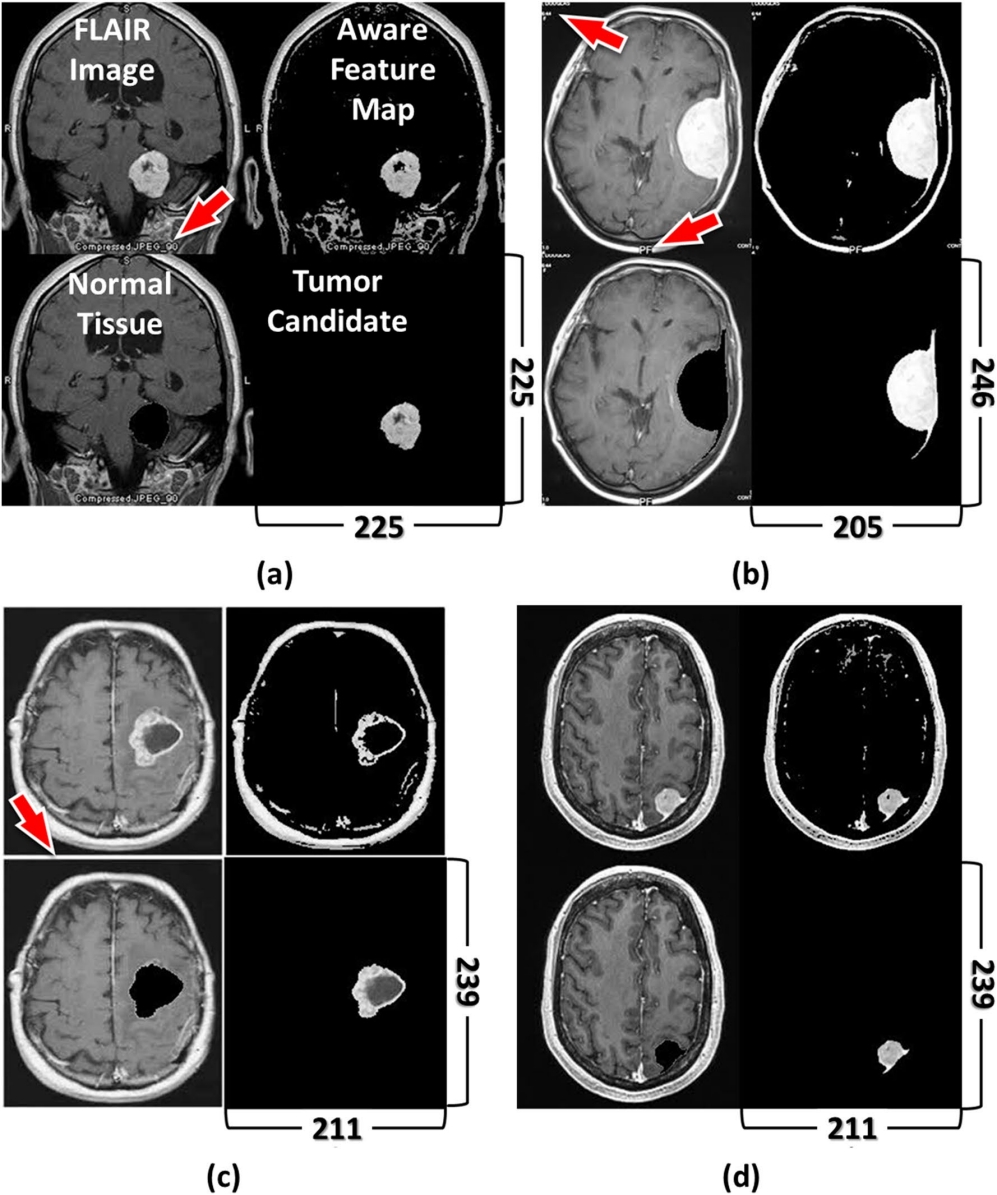
The performance of cases with interferences. As labeled in (**a**), the four sub-images of each panel indicate the FLAIR image (upper-left), the aware feature map (upper-right), normal tissue (lower-left), and the tumor candidate (lower-right). Image dimensions are next to the owning image in the lower right corner. The FLAIR images of (**a**) and (**b**) have notations added by radiologists (indicated by red arrows) and represent coronal and axial views, respectively. There is a white margin (indicated by a red arrow) in the original image of (**c**) and a relatively small tumor in (**d**). The CPU time for this small dataset ranges between 0.06 to 0.09 s. We acquired these images from an open-source dataset^33^.

To exhibit the ingenuity of the fDDFT and its advantage of using low-cost hardware, we deliberately chose a CPU-based operating system with a dual-core @ 3.8 GHz and 32 GB of memory. The CPU times for automatic brain tumor recognition and segmentation (see Step 4 of Fig. 2) under the DDFT and fDDFT frameworks were 10.4 and 0.05 s, respectively. It should emphasize that the conventional DDFT employed 4-thread parallel computations to overcome the inevitable high computational complexity from its PEDF estimation. The significant reduction in computational time consumption (about 208x) achieved by the fDDFT reveals its superiority over the previous version. We also investigated its capability on other types of images^33^, and Fig. 3 illustrates the relevant segmentation results. As shown in the upper-left corner of each panel, the input images exhibit different outlooks. Figures 3a and b are coronal and axial views of brain MR images with notations labeled by radiologists as red arrows indicate. Figure 3c has a white margin surrounding the MR image. Even with these interferences, the fDDFT still generates precise tumor segmentations. In addition, the fDDFT provides successful segmentation of small tumors, as shown in Fig. 3d.

### The feature-aware recognition and segmentation of three-dimensional brain tumor image datasets

To verify the fDDFT’s capability on pattern recognition and segmentation for high dimensional data structures, we exploited the Brain Tumor Segmentation Challenge 2020 (BraTS 2020) dataset^34–36^. The dataset comprises magnetic resonance imaging (MRI) scans with 369 training sets and corresponding labels. Each trial includes 155 slices with 240 pixels $\times$ 240 pixels in size and has multimodal images acquired from sequences of T1-weighted, T1 contrast-enhanced (T1-CE), T2-weighted, and T2 fluid-attenuated inversion recovery sequence (T2 FLAIR) volumes. Image labels comprise the necrotic and non-enhancing tumor core (NCR/NET), the GD-enhancing tumor (ET), the peritumoral edema (ED), and their union, i.e., the whole tumor (WT). Thus, we can treat this circumstance as 369 isolated systems. Each system has a dimension of $H\times W\times D=$ 240 grids $\times$ 240 grids $\times$ 155 grids and has specific compounds embedded in its owning soft tissue. Since the task now is to identify the locations and appearances of these compounds under the fDDFT framework, we only employed the T2 FLAIR datasets as inputs and considered the contribution of the WT image labels in this experiment.

Diverse intensity distributions and structural combinations of edema, necrosis, tumor cores, and brain tissues raise the difficulty in pattern recognition. It is like non-negligible defects doped within a compound and widens the original energy level. From the GNN’s perspective, it is similar to an induced subgraph of a complete graph that misses its edges and then loses the connection or similarity to other subgraphs. Meanwhile, intensity levels are inconsistent between MRI sequential slices or within trial scans due to different imaging protocols and apparatuses, which compelled us to amend the algorithm of similarity convergence. Despite all cases in Figs. 2 and 3 having obvious brain tumor patterns in their image representations, the searching task in three-dimensional brain tumor image datasets must first judge whether an MR slice has elements belonging to the brain tumor. The problems mentioned above would reduce the accuracy of similarity convergence and then obstruct the operations of pattern recognition and segmentation.

To reinforce the pattern recognition capability, we introduced LDF and HDF into the algorithm of similarity convergence. We replaced the subgraph group of PEDF with that of HDF ${\mathcal{H}}_{E}$ to find the candidate subgraph of tumors:

11 $${\mathcal{T}}_{C}=\left\{{\mathcal{T}}_{{S}_{i}}|{\mathcal{H}}_{E}\cap {\mathcal{T}}_{{S}_{i}}\notin \varnothing \right\}, i=\mathrm{1,2},\dots ,N.$$

**Figure 4:**
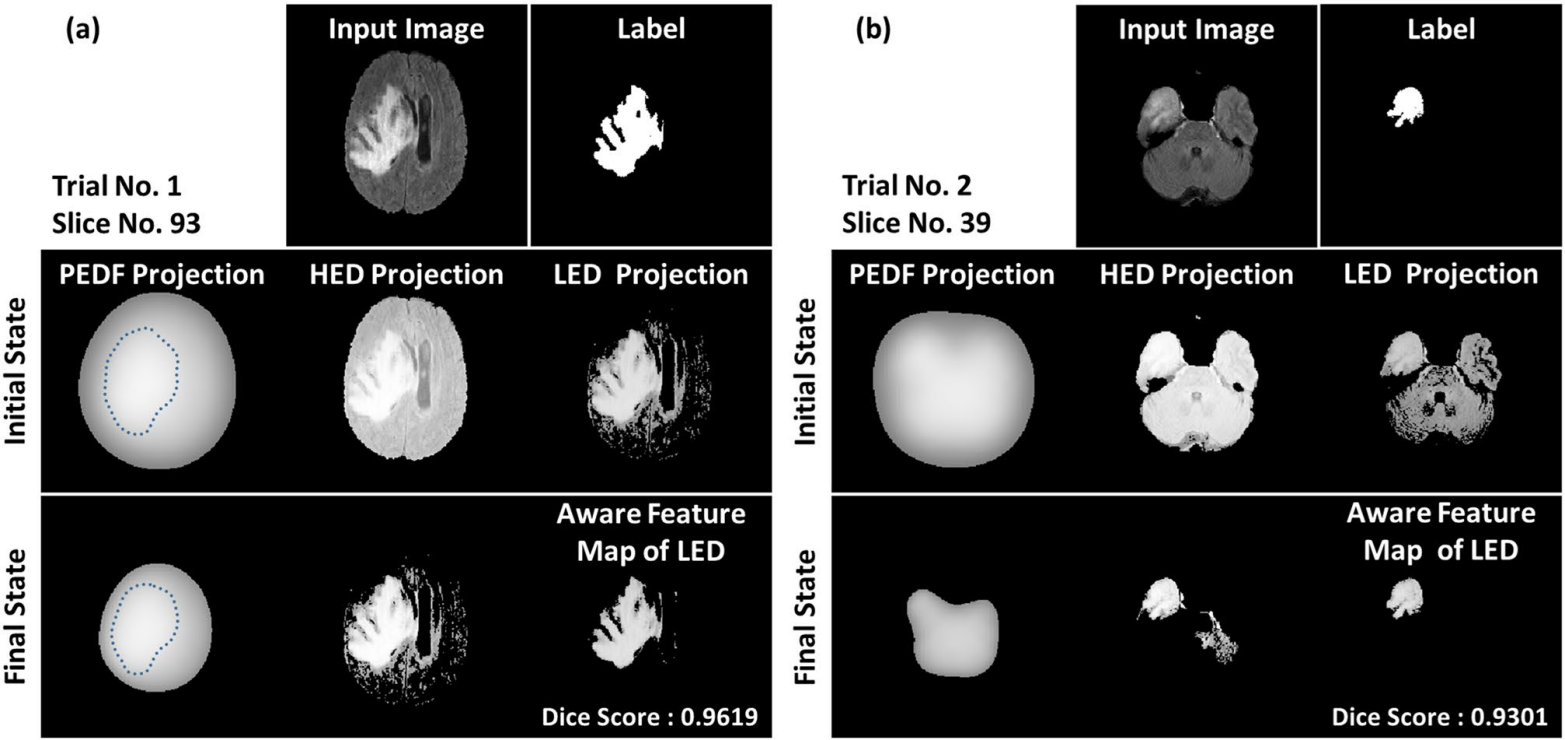
Demonstrations of the reinforced algorithm of similarity convergence. (**a**) and (**b**) exhibit different input figure representations and their corresponding projection area evolutions of functional landscapes. The former represents a general situation of similarity convergence, in which the PEDF projection, as expected, indicates the range with high similarity in the final state, as shown by the dashed lines. However, since the figure representation in case (**b**) has three discrete blocks, the biggest one attracts much attention in the PEDF estimation and products a deformed PEDF projection. Thus, we introduced the HED and LED projections to replace the original PEDF one to generate better outcomes for this kind of circumstance.

Figure 4 illustrates the evolutional landscapes of these functionals under the procedure of similarity convergence. We used the area of the 2-dimensional projection of the PEDF landscape as the criterion of reaching similarity convergence. Fermi normalization was employed to update these projection areas of functionals in the algorithm. The Fermi normalization truncates and saves areas with the top 50% energy levels in each round, prompting these areas to move toward the ranges with high similarity. The segmentation results in Fig. 4 reveal that introductions of the HED and LED projections raise the performance of brain tumor recognition compared to that of the original PEDF projection. The shapes of the PEDF projection in the initial and final states of Fig. 4a are similar, as indicated by the dashed lines, so its landscape suggests the range of high similarity as expected. However, the PEDF projection in the final state of Fig. 4b suffers a significant deformation compared to its shape in the initial state. There are three separate graphic blocks in the input image. Even though the PEDF projection also points to the high-similar range, it puts too much attention on the largest block. From the perspective of neural networks, the PEDF projection in the similarity convergence causes an overfitting on the feature map. Compared to the outcome of the PEDF projection, the use of HED and LED presents a better capability for brain tumor pattern recognition.

### Statistical results of feature-aware segmentation and limitations on low-featured subgraph recognition

**Figure 5:**
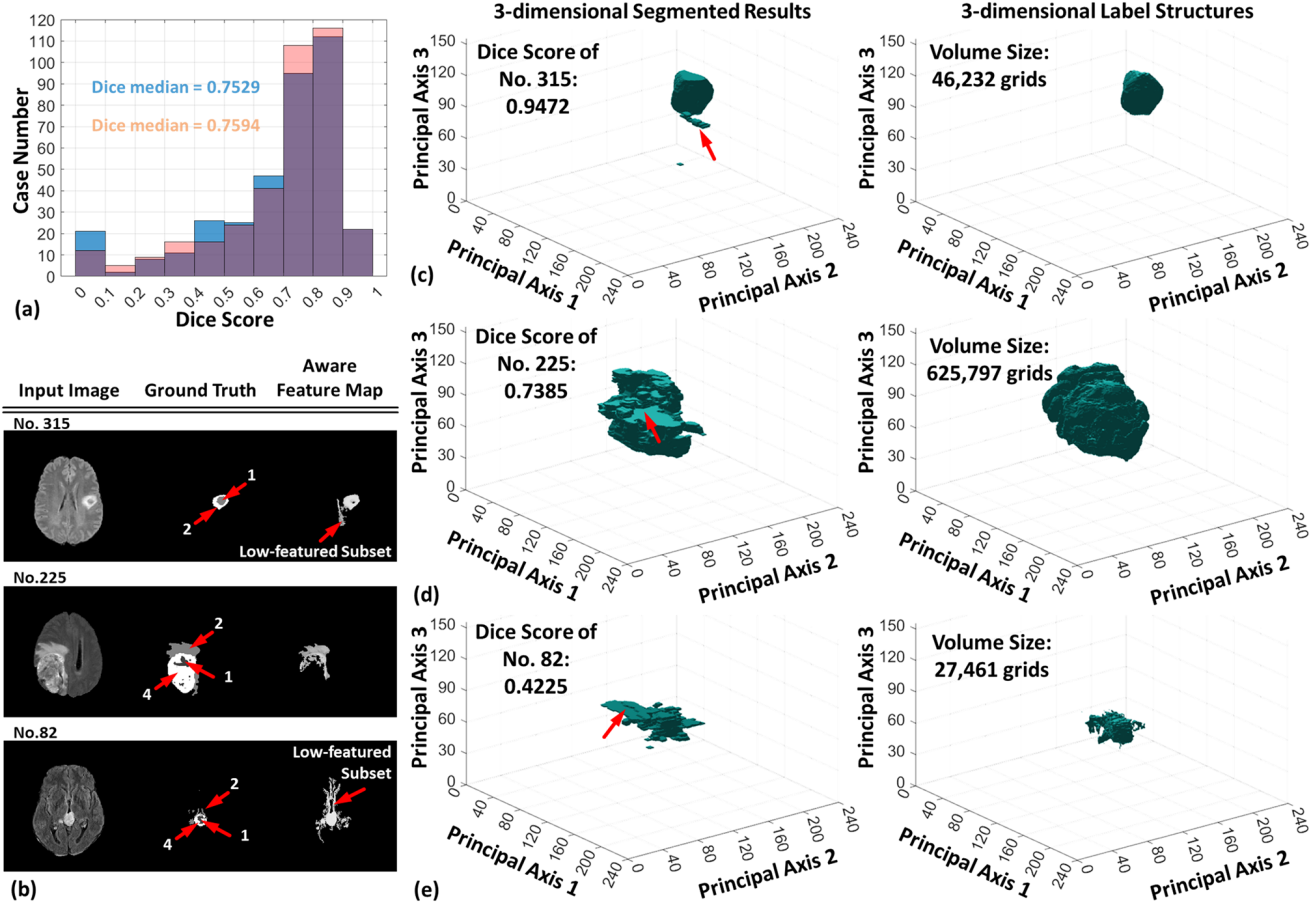
The accuracy distribution and structural analyses of representative cases. As illustrated in (**a**), the soft dice score distribution reaches the standard performance of conventional deep neural networks. The blue and pink bars in (**a**) represent the statistical calculations using values of the permissible smallest edge number of $3\pi \sqrt{{\Vert I\Vert }_{0}}/2$ and $\pi \sqrt{{\Vert I\Vert }_{0}}$, respectively. (**b**) exhibits the two-dimensional aware feature maps of three representative results. The numbers of 1, 2, and 4 represent labels of the necrotic and non-enhancing tumor core, the peritumoral edema, and the GD-enhancing tumor, respectively. (**c**), (**d**) and (**e**) present the representative reconstructed results of three-dimensional brain tumor images and their corresponding label structures.

Figure 5 illustrates the statistical results of dice score estimations and the three-dimensional reconstructions of representative brain tumors. To simplify the procedure of pixel connectivity, we only allowed the subgraphs with the smallest edge number exceeding the value of $3\pi \sqrt{{\Vert I\Vert }_{0}}/2$ to stay on the aware feature map of the LED, where ${\Vert I\Vert }_{0}$ is the ${\mathcal{l}}_{0}$ norm of an input image $I\in {\mathbb{R}}^{H\times W}$. The blue bars in Fig. 5a show the statistical distribution of the dice score for the whole BraTS 2020 set. The mean soft dice score of the unsupervised brain tumor image segmentation performed by aware feature maps is 0.6765. The quartiles are 0.5995, 0.7529, and 0.8402; the corresponding case numbers are 277, 185, and 92. The CPU time of fDDFT execution for each trial was about 1.76 s on average. The median dice score of the unsupervised brain tumor recognition and segmentation using the fDDFT has reached the standard performance of conventional deep neural networks^8,30^. To visualize unsupervised segmentation results and discuss the limitations, we present the results of three representative cases to elaborate on the interactions between low-featured subgraphs and aware features. Descriptions of how these situations are analogous to the behavior of impurities along the Fermi surface in a chaotic physical system are also provided. Figure 5b displays two-dimensional aware feature maps with low-featured subgraphs of the three cases. The numbers of 1, 2, and 4 markered on each ground truth represent NCR/NET, ED, and ET labels, respectively. With the highest soft dice score, case No. 315 has a low-featured subgraph connected to the tumor body because of the low heterogeneity between the brain tissue and tumor, resulting in a not-perfect dice score. It is similar to a few impurities occupied near the Fermi surface and had slight perturbation on energy levels. In Fig. 5c, we exhibit the reconstructed three-dimensional brain tumor of No. 315 and the label structure. The red arrow below the tumor indicates an undesired part formed by a low-featured subgraph embedded in the native aware feature map.

Cases 225 and 82 represent huge and tiny tumor bodies, respectively. Figure 5d and e illustrate their reconstructed results. The aware feature map of No. 225 shows an unsatisfying result. It misses the desired GD-enhancing tumor, shown in the middle panel of Fig. 5b. We deduce that pixels with low energy and weak pair connection, as shown in the input image, block out the feature extraction in the procedure of similarity convergence. It is like several low-energy impurities filled below the Fermi surface; they obstruct the electron transmission along the surface and thus cover the exertion of intrinsic material properties. Since giant tumors often produce a wide range of GD-enhancing tumor representations in MRI, low-energy pixels of these representations would obstruct unsupervised tumor pattern recognition, as shown in the reconstructed result of Fig. 5d. We also inspected the capability of pattern recognition and segmentation on tiny tumors. The low-featured subset of No. 82 displays a low heterogeneity between the tumor body and the thalamus, as shown in the bottom panel of Fig. 5b. Hence, the LDF could hardly distinguish their energy difference in the procedure of similarity convergence. This situation is analogous to the impurities occupied above the Fermi surface, and their induced carriers dominate the electron transmission within the conduction band. It also means that signals in the conduction band are mixed from the transmission and induced carriers. In other words, low heterogeneity would also significantly limit the capability of brain tumor pattern recognition and segmentation, as shown in Fig. 5e.

To avoid the specificity of the permissible smallest number interfering with the statistical result of the soft dice score, we also replaced the number with $\pi \sqrt{{\Vert I\Vert }_{0}}$. The pink bars in Fig. 5a exhibit the corresponding statistical outcomes. It should be noted that we only applied this number to those trials with soft dice scores below the mean value of the original distribution. In this additional experiment, the quartiles are 0.6344, 0.7594, and 0.8414. As shown in Fig. 5a, the case number over the dice score of 0.7 increased significantly, but the other cases moved to the range of low scores. Thus, these statistical measures did not lead to a significant increase in the average dice score. In other words, as complex as a chaotic physical system, it is difficult to describe the diverse modality of brain tumor images by controlling the permissible smallest edge number naively.

### The synergy between fDDFT and deep learning model

To inspect the synergic performance of applying the fDDFT to the conventional deep neural network, we also adapted the dimension-fusion U-Net (D-UNet)^37^. The hardware specifications were AMD Ryzen Threadripper PRO 3955WX 16-core CPU and an NVIDIA GeForce RTX 3090 GPU in this experiment. The initial learning rate was $1.5\times {10}^{-4}$, and the batch size and epoch were 16 and 60, respectively. We randomly picked 85% and 15% from the training set of BraTS 2020 as the training and validation sets. We also used the soft dice loss^30^ for loss function estimation and employed Adam as the optimizer in the training procedure. Table 1 lists the performance comparison, including the training time, inference time, and segmentation capability between the naïve D-Unet and the synergic method. In the synergic method, we used fDDFT on the whole dataset and fed it into the D-Unet as inputs. The total preprocessing time of fDDFT is about 14.4 min. The training and inference time was significantly reduced by 58% and 51%, respectively. Meanwhile, the whole segmentation scores of the synergic method are all superior to those of the naïve D-Unet. These results also match the GDL’s perspective^5^.

**Table 1:** Performance comparison between naïve D-UNet and the proposed method.

Model	Training time (s/epoch)	Inference time (ms/subject)	Dice score	
D-UNet	1490	3.33	WT	0.9367
			NCR/NET	0.7372
			ET	0.8040
			ED	0.8131
fDDFT + D-UNet	626 (− 58%)*	1.64 (− 51%)	WT	**0.9415**
			NCR/NET	**0.7996**
			ET	**0.8376**
			ED	**0.8626**

*Values in parentheses mean the increased rates compared to the D-UNet.

Significant values are in bold.

## Discussion

We propose the fast data density functional transform (fDDFT) for feature-aware unsupervised pattern recognition and segmentation based on the DFT configuration and the GDL architecture. Under the fDDFT framework, we create an AutoEncoder-assisted module to reduce the computational complexity of global convolutional operations, a skill of geometric stability to enhance the capability of pattern recognition, and a mechanism of similarity convergence to assist the feature selection. We also utilize three-dimensional brain tumor structures to imitate soft matter in a chaotic physical system and inspect unsupervised soft matter pattern recognition and segmentation capability. The performance of this framework achieves the standard requirement for conventional deep neural networks. The representative cases reflect ordinary circumstances in actual physical systems and thus point out the limitations of the fDDFT. Procedures of similarity convergence and pixel connectivity in the fDDFT have to face the challenges of low intensity, weak pair connection, and low heterogeneity.

Integrating the data structures from DFT and contemporary deep neural networks is another significant contribution of this article. Even though managing data structures under graphic architecture is still mainstream, the fDDFT regularizes and transforms these structures in the Euclidean space under the GDL architecture and GNN metrics. This strategy benefits the connection between the non-Euclidean and the grid spaces and reinforces the capability of data structural visualization for computational modeling applications.

On the other hand, we have to emphasize that using two-stage calculation to obtain better soft dice score distributions, as shown in Fig. 5a, is not permissible in deep learning. Deep learning methods pursue a direct way for lesion pattern recognition and segmentation because they assume the corresponding labels are not accessible, and the training procedures are time-consuming. Modifying important parameters in deep learning implies possible retraining of the whole model. Fortunately, the fDDFT can easily accommodate parameter rearrangement in any procedure of the model pipeline because of the short inference time of 2 s. The fDDFT can be a precursor of modern deep neural networks on feature selection and enhancement. The synergic experiment has validated that our proposed method can significantly improve the training time, inference time, and segmentation capability of neural networks. This technical advantage could benefit clinical investigations for clinicians to verify previous results, modify parameters, and obtain updated segmented outcomes.

### Supplementary Information

Supplementary Information.

## Data Availability

The BraTS 2020 dataset is publicly available (https://www.med.upenn.edu/cbica/brats2020/data.html). All other datasets that support this work are also available from the corresponding references. The Matlab code developed in this work is available in the Supplementary file.
