# Supplementary material for: Feature-aware unsupervised lesion segmentation for brain tumor images using fast data density functional transform
Source: Sci Rep. 2023 Aug 21;13:13582. doi: 10.1038/s41598-023-40848-5 (PMC10442428; doi:10.1038/s41598-023-40848-5)
Supplement: Supplementary file 1 — Supplementary Information. [file 41598_2023_40848_MOESM1_ESM.pdf]

Feature-aware unsupervised lesion segmentation for brain tumor images using fast data density functional transform

Shin-Jhe Huang[1,+], Chien-Chang Chen[1,+], Yamin Kao[1] & Henry Horng-Shing Lu[2,*]

[1] Geometric Data Vision Laboratory, Department of Biomedical Sciences and Engineering, National Central University, Taoyuan City 32001, Taiwan.

[2] Institute of Statistics, National Yang Ming Chiao Tung University, Hsinchu 30010, Taiwan.

[+]these authors contributed equally to this work

[*]Corresponding author: henryhslu@nycu.edu.tw.

Supplementary Code

```matlab
%% This MATLAB code demonstrates the segmented cases shown in Fig. 1 and 2.
%% The version of MATLAB OS environment is R2021a
%% The images can be downloaded from
https://www.mathworks.com/matlabcentral/fileexchange/55107-brain-mri-tumor-detection-and-classification?s_tid=prof_contriblnk

%% Initial conditions
    clear; close all; clc;
    timem = tic;

%% energy calculation
resize_fac = 0.125;     % Compression factor
plotidx = 1;

for i = 1
    tval = tic;
    im = double(imread(['.\MRI\',num2str(i),'.jpg']))./255;
    im = rgb2gray(im);
    [HH, WW] = size(im);
    Im_length = sum(im(im > 0.05));

    %%%%%% PEDF calculaton %%%%%%
    im_res = imresize(im, resize_fac);
    [H, W] = size(im_res);
    V = zeros(2*H-1, 2*W-1);
    V(H, W) = 1;
    dist_inv = 1 ./ bwdist(V,'euclidean');
    dist_inv(isinf(dist_inv)) = 0;
    V = CONV2FFT(im_res, dist_inv);
    V = imresize(V, [HH, WW]);

    %%%%%% KEDF calculation %%%%%%
    KED = 2*pi*im;

    %%%%%% gamma estimation %%%%%%
    gamma = 0.5*sum(V(:).*im(:))/sum(KED(:).*im(:));
    LED = -V * gamma + KED * gamma^2;

    %%%%%% LDF balance %%%%%%
    newLED = mean(LED(:));
    while mean(LED(:)) <= 0
```

```
    oldLED = mean(LED(:));
    gamma = gamma + 0.5 * newLED./oldLED;
    LED = -V * gamma + KED * gamma^2;
    newLED = mean(LED(:));
end
LDF = LED .* (LED > mean(LED(LED > 0)));
LDF_imag = im .* (LED > mean(LED(LED > 0)));

%%%%% Candidate Segmentation %%%%%
% subsets of LDF
CC = bwconncomp(LDF, 8);
numPixels = cellfun(@numel, CC.PixelIdxList);
numPixels(numPixels < pi*sqrt(Im_length)) = 0;
numPixels_idx = find(numPixels ~= 0);

% subsets of PEDF
while sum(V(:)) > Im_length * 0.5
    V = fermi(V);
    V = V .* (V > 0.5);
end
VMASK = find(V > 0);

mask_face = zeros(HH,WW);
for ii = numPixels_idx
    indexMAX = CC.PixelIdxList{1, ii};
    con1 = sum(ismember(indexMAX, VMASK));
    if con1
        mask_face_temp = zeros(HH,WW);
        mask_face_temp(indexMAX) = 1;
        mask_face_temp = imfill(mask_face_temp,'holes');
        mask_face = mask_face + mask_face_temp;
    end
end

mask_face = imfill(mask_face,'holes');
VI = ~mask_face .* im;
IV = mask_face .* im;

disp(['       estimated time : ',num2str(toc(tval)), 'sec.']);

if plotidx
```

```
            figure, mesh(V), view(-10, 60), axis ij, axis off;
            figure, mesh(KED), view(-10, 75), axis ij, axis off;
            figure, mesh(LED), view(-90, 0), axis ij, axis off;
            figure, imshow([im, LDF_imag; VI, IV],[]);
            figure, imshow(LDF_imag,[]);
            drawnow
      end

end

disp(['Total estimated time is : ', num2str(toc(timem)), 'sec.']);
```

```matlab
%% This MATLAB code demonstrates the unsupervised pattern
%% recognition and segmentation of BraTS 2020 dataset.
%% The version of MATLAB OS environment is R2021a
%% The dataset is available at https://www.med.upenn.edu/cbica/brats2020/data.html

%% Initial conditions
    clear; close all; clc;
    timem = tic;

%% energy calculation
resize_fac = 0.125;     % Compression factor
im3 = zeros(240, 240, 155);
im3d = zeros(240, 240, 155);
mask3d = im3d;
LDF3d = im3d;
HED3d = im3d;
dice = zeros(155,1);
numstream = 1 : 369;

timp = tic;

for nump = numstream
    for numc = 1 : 155
        im3(:, :, numc) = double(imread(['.\BraTS2020\patient_', num2str(nump),'\img_' ,
num2str(nump), '_3_', num2str(numc), '.png'])),/255;
        mask3d(:, :, numc) = double(imread(['.\BraTS2020\patient_',
num2str(nump),'\img_' , num2str(nump), '_5_', num2str(numc), '.png']));
         % second number : (t1', 't2', 'flair', 't1ce', 'seg' in order)
    end

    im3(im3 < 0.05) = 0;
    im3min = mean(im3(im3 > 0));

    disp(['     # of Patent ', num2str(nump)]);
    tval = tic;

    for numc = 1 : 155
        im = im3(:, :, numc);
        [HH, WW] = size(im);
        Im_length = sum(im > 0.05, 'all');
        Im_length_min = 1.5*pi*sqrt(Im_length);
```

```
if Im_length/HH/WW >= 0.1
    stats.im = regionprops(im>0.05, "BoundingBox");
    if numel(stats.im) > 0
        for sas = 1 : numel(stats.im)
            aa(sas,1) = stats.im(sas).BoundingBox(3);
            aa(sas,2) = stats.im(sas).BoundingBox(4);
        end
    else
        aa = [1 1];
    end
    aa = [max(aa(:,1)), max(aa(:,2))];

    im_mask = mask3d(:, :, numc);

    %%%%% PEDF calculaton %%%%%
    im_res = imresize(im, resize_fac);
    [H, W] = size(im_res);
    V = zeros(2*H-1, 2*W-1);
    V(H, W) = 1;
    dist_inv = 1 ./ bwdist(V,'euclidean');
    dist_inv(isinf(dist_inv)) = 0;
    V = CONV2FFT(im_res, dist_inv);
    V = imresize(V, [HH, WW]);

    %%%%% KEDF calculation %%%%%
    KED = 2*pi*im;

    %%%%% gamma estimation %%%%%
    gamma = 0.5*sum(V(:).*im(:))/sum(KED(:).*im(:));
    LED = -V * gamma + KED * gamma^2;

    %%%%% Geometric Stability of LDF %%%%%
    newLED = mean(LED(:));
    if ~isnan(newLED)
        while mean(LED(:)) <= 0
            oldLED = mean(LED(:));
            gamma = gamma + 0.5 * newLED./oldLED;
            LED = -V * gamma + KED * gamma^2;
            newLED = mean(LED(:));
        end
```

```
else
    LED = zeros(size(V));
end
HED = V * gamma + KED * gamma^2;

%%%%% Candidate Segmentation %%%%%
% subsets of PEDF
% Similarity Convergence
while sum(V(:)) > Im_length * 0.5
    HED = fermi(HED);
    HED = HED .* (HED > 0.5);
    LED = fermi(HED);
    LED = LED .* (LED > 0.5);
    V = fermi(V);
    V = V .* (V > 0.5);
end
LDF = bwareaopen(LED, round(Im_length_min));
sast = HED;
HED = bwareaopen(HED, round(Im_length_min));
VMASK = find(HED > 0);

% subsets of LDF
CC = bwconncomp(LDF, 8);
numPixels = cellfun(@numel, CC.PixelIdxList);

if (sum(numPixels) > Im_length * 0.5)
    numPixels(:) = 0;
else
    numPixels(numPixels < Im_length_min ) = 0;
end
numPixels_idx = find(numPixels ~= 0);

mask_face = zeros(HH,WW);
for ii = numPixels_idx
    indexMAX = CC.PixelIdxList{1, ii};
    con1 = sum(ismember(indexMAX, VMASK));
    if con1
        mask_face_temp = zeros(HH,WW);
        mask_face_temp(indexMAX) = 1;
        mask_face_temp = imfill(mask_face_temp,'holes');
        mask_face_temp = imerode(mask_face_temp, ones(5));
```

```
                    stats.mask = regionprops(mask_face_temp, "BoundingBox");
                    if ~isempty(stats.mask)
                    if stats.mask.BoundingBox(3) / aa(1) < 0.75 ...
                            && stats.mask.BoundingBox(4) / aa(2) < 0.75
                        mask_face_temp = imdilate( mask_face_temp, ones(5));
                        mask_face = mask_face + mask_face_temp;
                    end
                    end
                end
            end

            mask_face = imfill(mask_face,'holes');
            im3d(:, :, numc) =    mask_face;
            HED3d(:, :, numc) = sast .* HED;
            LDF3d(:, :, numc) = LED .* LDF;
            dice(numc) = 2*nnz(mask_face & im_mask)/(nnz(mask_face) + nnz(im_mask) +
1e-6);
        else
            dice(numc) = 1;
            im3d(:, :, numc) = zeros(HH,WW);
            LDF = zeros(HH,WW);
            LED = LDF;
            HED = zeros(HH,WW);
            HED3d(:, :, numc) = HED;
            LDF3d(:, :, numc) = LDF;
            mask_face = zeros(HH,WW);
            V = mask_face;
            im_mask = mask3d(:, :, numc);
        end

        if dice(numc) > 0.5 && sum(LDF(:)) > 0 && (length(numstream) < 2)
            figure(1000*nump + numc),
            imshow([im sast.*HED LED.*LDF], [])
            text(0, HH+10, num2str(dice(numc)))
            drawnow
        end

    end

    disp(['        estimated time : ',num2str(toc(tval)), 'secs.']);
```

```
        energyball = HED3d;
        CCim3d = bwconncomp(im3d);
        energyMean = zeros(numel(CCim3d.PixelIdxList),1);
        for sq = 1 : numel(CCim3d.PixelIdxList)
            energyMean(sq) = mean(energyball(CCim3d.PixelIdxList{1,sq}));
        end
        [~, idxenergy] = max(energyMean);
        im3dPixels = cellfun(@numel, CCim3d.PixelIdxList);
        [~, idx3d] = max(im3dPixels);
        im3dr = zeros(size(im3d));
        if ~isempty(idxenergy) && ~isempty(idx3d)
            im3dr(CCim3d.PixelIdxList{1, idxenergy}) = 1;
            im3dr(CCim3d.PixelIdxList{1, idx3d}) = 1;
        end

    dice_total(nump) = 2*nnz(im3dr & mask3d)/(nnz(im3dr) + nnz(mask3d) + 1e-6);
    disp(dice_total(nump))
    disp(' ')

end
disp(['       estimated time : ',num2str(toc(timp) ./ 60), 'mins.']);
disp(['Dice mean = ', num2str(mean(dice_total(:)))]);
[aa, bb] =   max(dice_total(:));
disp(['Dice max = ', num2str(aa), ', @ patient # ', num2str(bb)]);
[aa, bb] =   min(dice_total(:));
disp(['Dice min = ', num2str(aa), ', @ patient # ', num2str(bb)]);

dice_total_prev = dice_total;

if length(numstream) < 2
    figure, isosurface(im3dr), axis([0 HH 0 WW 0 155]), grid on, axis ij
    set(gca, 'FontSize', 14), xticks(0:40:240), yticks(0:40:240), zticks(0:30:150) ,
    figure, isosurface(mask3d > 0), axis([0 HH 0 WW 0 155]), grid on, axis ij
    set(gca, 'FontSize', 14), xticks(0:40:240), yticks(0:40:240), zticks(0:30:150),
end

%% energy calculation: 2nd round
resize_fac = 0.125;     % Compression factor
im3 = zeros(240, 240, 155);
im3d = zeros(240, 240, 155);
mask3d = im3d;
```

```
LDF3d = im3d;
HED3d = im3d;
dice = zeros(155,1);
numstream = find(dice_total < mean(dice_total_prev));

for nump = numstream
    for numc = 1 : 155
        im3(:, :, numc) = double(imread(['.\BraTS2020\patient_', num2str(nump),'\img_' ,
num2str(nump), '_3_', num2str(numc), '.png']))./255;
        mask3d(:, :, numc) = double(imread(['.\BraTS2020\patient_',
num2str(nump),'\img_' , num2str(nump), '_5_', num2str(numc), '.png']));
         % second number : (t1', 't2', 'flair', 't1ce', 'seg' in order)
    end

    im3(im3 < 0.05) = 0;
    im3min = mean(im3(im3 > 0));

    disp(['     # of Patent ', num2str(nump)]);
    tval = tic;

    for numc = 1 : 155
        im = im3(:, :, numc);
        [HH, WW] = size(im);
        Im_length = sum(im > 0.05, 'all');
        Im_length_min = 1*pi*sqrt(Im_length);

        if Im_length/HH/WW >= 0.1
            stats.im = regionprops(im>0.05, "BoundingBox");
            if numel(stats.im) > 0
                for sas = 1 : numel(stats.im)
                    aa(sas,1) = stats.im(sas).BoundingBox(3);
                    aa(sas,2) = stats.im(sas).BoundingBox(4);
                end
            else
                aa = [1 1];
            end
            aa = [max(aa(:,1)), max(aa(:,2))];

            im_mask = mask3d(:, :, numc);

            %%%%%% PEDF calculaton %%%%%%
```

```
im_res = imresize(im, resize_fac);
[H, W] = size(im_res);
V = zeros(2*H-1, 2*W-1);
V(H, W) = 1;
dist_inv = 1 ./ bwdist(V,'euclidean');
dist_inv(isinf(dist_inv)) = 0;
V = CONV2FFT(im_res, dist_inv);
V = imresize(V, [HH, WW]);

%%%%% KEDF calculation %%%%%
KED = 2*pi*im;

%%%%% gamma estimation %%%%%
gamma = 0.5*sum(V(:).*im(:))/sum(KED(:).*im(:));
LED = -V * gamma + KED * gamma^2;

%%%%% Geometric Stability of LDF %%%%%
newLED = mean(LED(:));
if ~isnan(newLED)
    while mean(LED(:)) <= 0
        oldLED = mean(LED(:));
        gamma = gamma + 0.5 * newLED./oldLED;
        LED = -V * gamma + KED * gamma^2;
        newLED = mean(LED(:));
    end
else
    LED = zeros(size(V));
end
HED = V * gamma + KED * gamma^2;

%%%%% Candidate Segmentation %%%%%
% subsets of PEDF
% Similarity Convergence
while sum(V(:)) > Im_length * 0.5
    HED = fermi(HED);
    HED = HED .* (HED > 0.5);
    LED = fermi(HED);
    LED = LED .* (LED > 0.5);
    V = fermi(V);
    V = V .* (V > 0.5);
end
```

```
        LDF = bwareaopen(LED, round(Im_length_min));
        sast = HED;
        HED = bwareaopen(HED, round(Im_length_min));
        VMASK = find(HED > 0);

        % subsets of LDF
        CC = bwconncomp(LDF, 8);
        numPixels = cellfun(@numel, CC.PixelIdxList);

        if (sum(numPixels) > Im_length * 0.5)
            numPixels(:) = 0;
        else
            numPixels(numPixels < Im_length_min ) = 0;
        end
        numPixels_idx = find(numPixels ~= 0);

        mask_face = zeros(HH,WW);
        for ii = numPixels_idx
            indexMAX = CC.PixelIdxList{1, ii};
            con1 = sum(ismember(indexMAX, VMASK));
            if con1
                mask_face_temp = zeros(HH,WW);
                mask_face_temp(indexMAX) = 1;
                mask_face_temp = imfill(mask_face_temp,'holes');
                stats.mask = regionprops(mask_face_temp, "BoundingBox");
                if stats.mask.BoundingBox(3) / aa(1) < 0.75 ...
                        && stats.mask.BoundingBox(4) / aa(2) < 0.75
                    mask_face = mask_face + mask_face_temp;
                end
            end
        end

        mask_face = imfill(mask_face,'holes');
        im3d(:, :, numc) =   mask_face;
        HED3d(:, :, numc) = sast .* HED;
        LDF3d(:, :, numc) = LED .* LDF;
        dice(numc) = 2*nnz(mask_face & im_mask)/(nnz(mask_face) + nnz(im_mask) +
1e-6);
    else
        dice(numc) = 1;
        im3d(:, :, numc) = zeros(HH,WW);
```

```
            LDF = zeros(HH,WW);
            LED = LDF;
            HED = zeros(HH,WW);
            HED3d(:, :, numc) = HED;
            LDF3d(:, :, numc) = LDF;
            mask_face = zeros(HH,WW);
            V = mask_face;
            im_mask = mask3d(:, :, numc);
        end

        if dice(numc) < 0.5 && sum(LDF(:)) > 0 && (length(numstream) < 2)
            figure(1000*nump + numc),
            imshow([im sast.*HED LED.*LDF], [])
            text(0, HH+10, num2str(dice(numc)))
            drawnow
        end

    end

    disp(['      estimated time : ',num2str(toc(tval)), 'secs.']);

        energyball = HED3d + LDF3d;
        CCim3d = bwconncomp(im3d);
        energyMean = zeros(numel(CCim3d.PixelIdxList),1);
        for sq = 1 : numel(CCim3d.PixelIdxList)
            energyMean(sq) = mean(energyball(CCim3d.PixelIdxList{1,sq}));
        end
        [~, idxenergy] = max(energyMean);
        im3dPixels = cellfun(@numel, CCim3d.PixelIdxList);
        [~, idx3d] = max(im3dPixels);
        im3dr = zeros(size(im3d));
        if ~isempty(idxenergy) && ~isempty(idx3d)
            im3dr(CCim3d.PixelIdxList{1, idxenergy}) = 1;
            im3dr(CCim3d.PixelIdxList{1, idx3d}) = 1;
        end
    dice_total(nump) = 2*nnz(im3dr & mask3d)/(nnz(im3dr) + nnz(mask3d) + 1e-6);
    disp(dice_total(nump))
    disp(' ')

end
disp(['      estimated time : ',num2str(toc(timp) ./ 60), 'mins.']);
```

```
disp(['Dice mean = ', num2str(mean(dice_total(:)))]);
[aa, bb] =   max(dice_total(:));
disp(['Dice max = ', num2str(aa), ', @ patient # ', num2str(bb)]);
[aa, bb] =   min(dice_total(:));
disp(['Dice min = ', num2str(aa), ', @ patient # ', num2str(bb)]);

if length(numstream) < 2
    figure, isosurface(im3dr), axis([0 HH 0 WW 0 155]), grid on, axis ij
    set(gca, 'FontSize', 14), xticks(0:40:240), yticks(0:40:240), zticks(0:30:150) ,
    figure, isosurface(mask3d > 0), axis([0 HH 0 WW 0 155]), grid on, axis ij
    set(gca, 'FontSize', 14), xticks(0:40:240), yticks(0:40:240), zticks(0:30:150),
end

%%
figure(99), histogram(dice_total_prev, 10, 'FaceAlpha',0.7), set(gca, 'FontSize', 16),
xticks(0: 0.1: 1), yticks(0: 10: 120), grid on;
text(0.1, 85, ['Dice Median = ', num2str(round(median(dice_total_prev(:)), 4))], 'FontSize', 16)
figure(99), hold on,
histogram(dice_total, 10, 'FaceAlpha', 0.3, 'FaceColor','r'),
set(gca, 'FontSize', 16), xticks(0: 0.1: 1), yticks(0: 10: 120), grid on;
text(0.1, 65, ['Dice Median = ', num2str(round(median(dice_total(:)), 4))], 'FontSize', 16),
drawnow

%%
disp([prctile(dice_total_prev,25), prctile(dice_total_prev,50), prctile(dice_total_prev,75)])
disp([prctile(dice_total,25), prctile(dice_total,50), prctile(dice_total, 75)])

disp([sum(dice_total >= prctile(dice_total,25)), ...
    sum(dice_total >= prctile(dice_total,50)), ...
    sum(dice_total >= prctile(dice_total,75))])
```

```
%% Function of Fermi Normalization
%% The version of MATLAB OS environment is R2021a
function I = fermi(I)
      meanI = mean(I(I>0));
      stdI = std(I(I>0));
      if ~isnan(meanI) && ~isnan(stdI)
            I = 1./(exp((meanI - I)./stdI) + 1);
      end
end
```

```
%% Function of Global Convolutional Operations
%% The version of MATLAB OS environment is R2021a
function C = CONV2FFT(A,B)
   [Ar,Ac] = size(A);
   [Br,Bc] = size(B);

   if any(any(imag(A))) || any(any(imag(B)))
         C = ifft2(fft2(A,Ar + Br - 1,Ac + Bc - 1).*fft2(B,Ar + Br - 1,Ac + Bc - 1));
   else
         C = real(ifft2(fft2(A,Ar+Br-1,Ac+Bc-1).*fft2(B,Ar+Br-1,Ac+Bc-1)));
   end
   r = ((Br-1)+mod((Br-1),2))/2;
   c = ((Bc-1)+mod((Bc-1),2))/2;
   C = C(r+1:r+Ar,c+1:c+Ac);
end
```
